# Electromyography- and Bioimpedance-Based Detection of Swallow Onset for the Control of Dysphagia Treatment

**DOI:** 10.3390/s24206525

**Published:** 2024-10-10

**Authors:** Benjamin Riebold, Rainer O. Seidl, Thomas Schauer

**Affiliations:** 1 Control Systems Group, Technische Universität Berlin, Einsteinufer 17, 10587 Berlin, Germany; riebold@tu-berlin.de; 2 Clinic for Ear, Nose and Throat Medicine, Unfallkrankenhaus Berlin (UKB), Warener Str. 7, 12683 Berlin, Germany; rainer.seidl@ukb.de

**Keywords:** dysphagia, swallow onset detection, biofeedback, functional electrical stimulation, machine learning

## Abstract

Several studies support the benefits of biofeedback and Functional Electrical Stimulation (FES) in dysphagia therapy. Most commonly, adhesive electrodes are placed on the submental region of the neck to conduct Electromyography (EMG) measurements for controlling gamified biofeedback and functional electrical stimulation. Due to the diverse origin of EMG activity at the neck, it can be assumed that EMG measurements alone do not accurately reflect the onset of the pharyngeal swallowing phase (onset of swallowing). To date, no study has addressed the timing and detection performance of swallow onsets on a comprehensive database including dysphagia patients. This study includes EMG and BioImpedance (BI) measurements of 41 dysphagia patients to compare the timing and performance in the Detection of Swallow Onsets (DoSO) using EMG alone versus combined BI and EMG measurements. The latter approach employs a BI-based data segmentation of potential swallow onsets and a machine-learning-based classifier to distinguish swallow onsets from non-swallow events. Swallow onsets labeled by an expert serve as a reference. In addition to the F1 score, the mean and standard deviation of the detection delay regarding reference events have been determined. The EMG-based DoSO achieved an F1 score of 0.289 with a detection delay of 0.018 s ± 0.203 s. In comparison, the BI/EMG-based DoSO achieved an F1 score of 0.546 with a detection delay of 0.033 s ± 0.1 s. Therefore, the BI/EMG-based DoSO has better timing and detection performance compared to the EMG-based DoSO and potentially improves biofeedback and FES in dysphagia therapy.

## 1. Introduction

### 1.1. Swallowing and Dysphagia

The ability to swallow is a body function of great importance. Dysphagia is an impairment of the swallowing process that severely reduces the quality of life and health status of patients. Dysphagia therapy is a time-consuming and demanding process for clinicians and patients. Manual treatment methods aim to enable safe food intake and help patients regain regular swallowing functions.

Worldwide, strokes occur in fifteen million people each year [1]. Almost 42% of stroke patients develop dysfunctional swallowing [2], and 33% aspirate, leading to pneumonia in 50% of these cases [1]. The mortality rate of patients with dysphagia causing aspiration is 18.9% [3]. Head and neck cancer treatment includes surgery, radiotherapy, and chemotherapy. Surgical interventions cause specific anatomical or neurological insults and likely result in specific swallow impairments. Radiotherapy and chemotherapy introduce several side effects, such as loss of appetite, mucous membrane inflammation (mucositis), and dysphagia [4]. The prevalence of dysphagia after treatment for head and neck cancer ranges from 33.0% to 71.0% [5].

Matsuo et al. [6] describe four swallowing phases, the preparatory oral phase, the propulsive oral phase, the pharyngeal phase, and the esophageal phase. During the oral preparatory phase, jaw and tongue movements masticate the food and mix it with saliva to produce a swallowing-appropriate bolus consistency. The tongue and soft palate seal the access to the pharynx for airway protection. After bolus preparation, the tongue transports the bolus backward to the pharynx in the propulsive oral phase.

The pharyngeal swallow phase is a rapid, reflexive sequence of actions to protect the airway and open the upper esophageal sphincter, giving the bolus passage to the esophagus. The soft palate and constrictor wall muscles close the access to the nasopharynx. The tongue base pushes the bolus to the pharynx, and the contraction of muscles in the pharyngeal wall forces the bolus downwards, triggering the excursion of the hyoid and larynx for epiglottis closure. Relaxation of the cricopharyngeus muscle enables the opening of the upper esophageal sphincter before laryngeal elevation. A complex contraction of suprahyoid and thyrohyoid muscles elevates the larynx and opens the upper esophageal sphincter. The tongue and the pharyngeal constrictor muscles introduce pressure on the bolus and push it into the upper esophageal sphincter. In the esophageal phase, the bolus arrives in the esophagus and is moved toward the stomach by peristaltic contractions of the esophagus.

**Figure 1:**
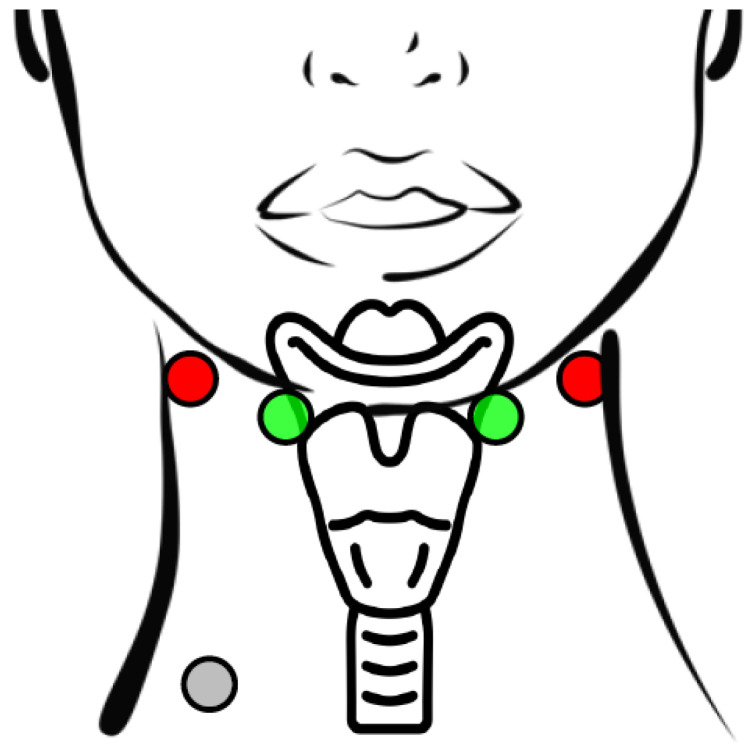
Electrode placement of the four-electrode setup with an additional reference electrode. The current electrodes (red) placed on sternocleidomastoideus close to the ear introduce a sinusoidal current of 50 kHz. The measurement electrodes (green) placed on each side of the larynx measure the voltage over the enclosed tissue. The reference electrode (gray) is used to suppress common-mode disturbances.

In this study, we employ BI and EMG measurements recorded with the PhysioSense device developed by Nahrstaedt et al. [7], utilizing a four-electrode setup, originally proposed by Yamamoto et al. [8], extended with a reference electrode. Figure 1 illustrates the electrode positions. The electrodes placed on the sternocleidomastoideus close to the ear on both sides of the neck inject a current, and two electrodes placed laterally to the larynx between the hyoid bone and the thyroid cartilage measure the voltage over the enclosed tissue. The bioimpedance is the absolute value of the transfer impedance at a frequency of 50 kHz regarding the voltage measured over the larynx and the inserted sinusoidal current.

**Figure 2:**
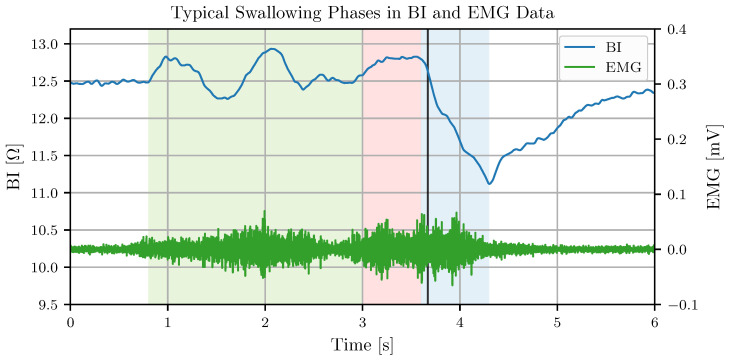
BI and EMG measurement of a saliva swallow. The swallow preparation phase (green background) shows some variation in the BI data caused by tongue movements from collecting saliva. The oral swallowing phase (red background) displays a small peak in the BI data, which continuously transitions to the BI swallow valley caused by the larynx elevation during the pharyngeal swallowing phase (blue background). The vertical line defines the time of the swallow onset marked by an expert, shortly after the start of the pharyngeal swallowing phase.

Figure 2 illustrates the typically observed BI valley of a swallow, coinciding usually with an active EMG period. The BI and EMG data in Figure 2 are cleaned with the preprocessing procedures presented in Section 2.4. Tongue and jaw movements in the oral swallowing phase introduce EMG activity and some variation in the BI data. In the propulsive oral swallowing phase, tongue movements transport the bolus to the back of the throat and the hyoid burst puts an initial force on the larynx and upper esophageal spincter [9]. These movements coincide with a small peak in the bioimpedance that precedes the BI swallow valley. The elevation of the larynx and the contraction of the pharynx cause a rapid BI drop. Once the pharynx relaxes and the larynx returns to its resting position, the BI data returns to the level before the swallow. Simultaneous Videofluoroscopic Swallowing Studies (VFSS) and BI/EMG recordings showed a high correlation of BI with the movement of the larynx and hyoid bone [10].

### 1.2. Biofeedback and FES for Dysphagia Therapy

Biofeedback is a technique to convert physiological processes into a representation in another modality. The visual, auditory, or haptic representation enables a patient to gain voluntary control over the physiological processes, which proceed automatically under healthy conditions. Feedback learning supports operant conditioning by providing information and motivation about the progress in physiological abilities and generates awareness about unconscious processes [11].

The majority of studies concerning biofeedback in dysphagia therapy employ electromyography (EMG) measurements with electrodes placed on the submental region. The most basic biofeedback approaches visualize the EMG or the EMG envelope on a screen to increase strength [12] and voluntary timing [13] of swallowing and to practice the Mendelsohn maneuver [14], the effortful swallow [15,16,17], or the volitional laryngeal vestibule closure [18]. More sophisticated techniques utilize onsets of EMG activity to control an avatar in gamified biofeedback [19,20,21]. Both approaches intend to improve the strength, duration, or timing of swallowing and increase motivation. In addition, Li et al. [22,23] employed an accelerometer placed on the larynx to trigger gamified biofeedback. Kwong et al. [24] showed the advantage of ultrasound biofeedback in learning the Mendelsohn maneuver compared to EMG-based biofeedback.

Nasal pressure [25] and the sound of Eustachian tube opening [26] have been used in recent studies with healthy adults as potential new non-invasive measurements for the characterization of the swallowing process. An assessment of these alternative measurements for biofeedback or triggered FES with dysphagia patients is still pending.

To date, meta-studies have found little definitive evidence of the benefit of most methods employed in dysphagia therapy [27,28]. Speyer et al. [28] reported studies supporting significant evidence with large effect sizes only for the Shaker exercise, the chin-tuck against resistance exercise, and expiratory muscle strength training. Moreover, none of the available studies concerning biofeedback in dysphagia therapy proved the benefit of the proposed methods [29,30].

Generally, electrical stimulation applies a current to nerves or muscle fibers through transcutaneous or percutaneous electrodes. The pulse duration, frequency, form, and amplitude define the stimulation current [31]. Functional electrical stimulation introduces action potentials in motor neurons to activate the connected muscle and cause a potent muscle contraction, resulting in a functional movement. Therefore, FES enables neuro-prostheses by restoring impaired motor functions. Usually, feedback control techniques adapt the stimulation current to compensate for unknown effects, such as the individual state of fatigue and voluntary contribution of the patients [32].

Several studies investigated triggered FES to support the swallowing process. Leelamanit et al. [33] achieved an improvement in swallow functions in 20 out of 23 dysphagia patients by administering triggered FES with adhesive electrodes to the thyrohyoid muscle. Burnett et al. [34] employed triggered FES to the mylohyoid, thyrohyoid, and thyrohyoid muscles with needle electrodes causing an up to 50% increase in larynx elevation compared to swallows without FES. In a follow-up study, Burnett et al. [35] showed that voluntary muscle activation is not reduced by triggered FES. Therefore, triggered FES has the potential to support swallowing [35]. Humbert et al. [36] applied transcutaneous FES at ten electrode positions and achieved an excursion of the larynx with two positions and an excursion of the hyoid with one position. Nahrstaedt et al. [37] reported a greater amplitude and velocity of the larynx excursion for triggered FES in a pilot study including a single dysphagia patient. Schultheiss et al. [38] studied the effect of triggered FES with multiple electrode setups on the amplitude and velocity of the larynx excursion in healthy subjects. They reported increased amplitude and velocity of the larynx excursion for most subjects and reduced amplitude and velocity of the larynx excursion for some subjects. Li et al. [23] measured a significantly shorter duration of the propulsive oral phase from the active EMG onset to the onset of acceleration caused by laryngeal excursion for triggered FES compared to swallows without FES in healthy subjects.

Hadley et al. [39] provoked a much greater excursion of the larynx by stimulating the hypoglossal nerve compared to stimulating the mylohyoideus, geniohyoideus, thyrohyoideus, and genioglossus with hook electrodes in five dogs and concluded that there was great potential for dysphagia management in humans. Tyler [40] called the absence of a robust trigger method as one of the greatest challenges for developing neuro-prostheses for dysphagia management.

### 1.3. Summary and Article Outline

Despite promising approaches presented in the literature concerning dysphagia therapy, most methods, including biofeedback, lack clear supporting evidence based on randomized controlled studies with numerous participants [28,29]. The studies on triggered FES in dysphagia therapy suggest great potential for dysphagia management as well. Triggered FES and some biofeedback methods require the detection of swallow onsets in real-time.

To date, techniques for swallow onset detection have employed EMG measurements at the throat [19,23,33], a combination of EMG and BI measurements [37], and measurement of the acceleration of the larynx [22,23]. EMG measurements taken at the throat usually show activation during swallow preparation [37]. Onsets of active EMG occur on average 0.5 s before the reflexive oral swallowing phase [23]. All reviewed techniques for the detection of swallow onsets require manual parameter tuning and lack a systematic analysis of the detection quality and timing using a comprehensive database including dysphagia patients.

We postulate that the success of biofeedback training and FES support of larynx elevation can be improved when the triggering occurs close to the onset of the pharyngeal phase. Triggering FES or presenting biofeedback during the bolus preparation in the oral phase is considered misleading and counterproductive. Hypoglossal nerve stimulation for dysphagia management in a neuro-prostheses requires robust detection of swallow onsets [40] even in everyday use.

Accordingly, we utilize a combination of EMG and BI data to detect swallow onsets coinciding with the start of the pharyngeal swallowing phase. Employing machine-learning methods omits manual parameter tuning in the proposed BI/EMG-based DoSO, while all single-sensor technologies are sensitive to non-swallowing activities such as speaking, chewing, and head and neck movements, the combination of different sensors is likely to decrease the false-positive rate in DoSO, especially in non-clinical environments. Using a comprehensive database with swallows and non-swallow events, an analysis of the detection quality and timing shows the advantage of the machine-learning-based approach using a BI/EMG-based approach compared to the standard threshold-based approach using EMG. The evaluation and optimization of the EMG-based and BI/EMG-based DoSO employs existing recordings of BI and EMG data. All algorithms presented in the article are suited for the online processing of BI and EMG data streams.

## 2. Materials and Methods

### 2.1. Database

The database of this study consists of four data series covering specific aspects of BI and EMG measurements in swallowing recorded with the PhysioSense device introduced by Nahrstaedt et al. [7]. The doctoral thesis of Holger Nahrstaedt [10] utilized the same database, and the doctoral thesis of Corinna Schultheiss [41] contains more detailed information about the included subjects.

The investigators labeled data segments containing swallows and movements during the recording procedure. Developing the real-time detection of swallow onsets demands annotations at the start of the pharyngeal swallowing phase. Therefore, an experienced examiner retrospectively added annotations at the start of the BI drop (Figure 2) providing references of swallow onset times. Table 1 contains an overview of the main features of data series I to IV.

#### 2.1.1. Data Series I

The study investigated the separability of swallows and head movements in BI and EMG measurements. Additionally, the data series contains swallows of boluses with varying electric conductivity and consistency to investigate the effect on BI measurements. The study included twenty healthy subjects divided in five subgroups. The first group swallowed 20 mL of water in a specific head position and performed head, yaw, and tongue movements, and speaking. The second group swallowed saliva and ten portions of liquid, each with a volume of 20 mL. The third group swallowed boluses of different consistencies, including saliva, 5 g of yogurt, and bread. The subjects of group four swallowed 50 mL, 10 mL, 20 mL, and 30 mL of water. The fifth group swallowed liquids with different electric conductivities.

#### 2.1.2. Data Series II

This data series examined the repeatability of the BI and EMG measurements with respect to electrode positioning. The study included fifteen subjects. Ten subjects provided four measurements conducted with slightly varying electrode positions recorded during a single day. All subjects participated in at least two measurements per day, repeated within four successive days. The subjects swallowed 200 mL of water at their own pace in a measurement session.

#### 2.1.3. Data Series III

The study comprises measurements executed by four investigators on nine subjects to investigate the reliability of the BI/EMG measurement method concerning different investigators. Therefore, each investigator placed the electrodes on the subject’s throat to prepare the measurements.

#### 2.1.4. Data Series IV

The data series includes measurements of 41 dysphagia patients. A total of 24 patients suffered from a neurological condition, and 17 patients from head-, ear-, nose-, and throat-related disorders. Depending on their condition and abilities, the patients swallowed saliva, dyed water, green jelly, and bread. An endoscopic examination, performed by a trained physician, accompanied the BI and EMG measurements to ensure the correct labeling of the swallows.

### 2.2. Assignment of Class Labels

The evaluation of a swallow onset detection requires a procedure to assign the potential swallow onsets determined at times of interest

(1) $$\begin{array}{cc} t_{i}^{\mathrm{TI}} & =\left(t_{1}^{\mathrm{TI}},\ldots ,t_{I}^{\mathrm{TI}}\right) \end{array}$$

with i=1,2,…,I being the reference times

(2) $$\begin{array}{cc} t_{r}^{\mathrm{RT}} & =\left(t_{1}^{\mathrm{RT}},\ldots ,t_{R}^{\mathrm{RT}}\right) \end{array}$$

with r=1,2,…,R annotated by an expert. Furthermore, training and testing a classifier for swallow onset detection demands a labeled data set

(3) $$\begin{array}{c} D=\{\left(y_{i},x_{i},s_{i}\right)\} \end{array}$$

consisting of binary class labels $y_{i}\in \left\{0,1\right\}$, feature vectors $x_{i}\in R^{J}$ with *J* features, and a subject index $s_{i}\in N^{+}$.

Calculating the absolute value differences

(4) $$\begin{array}{cc} \Delta t_{i} & =\left|t_{i}^{\mathrm{TI}}-t_{r}^{\mathrm{RT}}\right|\;\mathrm{with}\;i=1,2,\ldots ,I\;\mathrm{and}\;r=\mathrm{const}. \end{array}$$

between the times $t_{i}^{\mathrm{TI}}$ and a reference time $t_{r}^{\mathrm{RT}}$ delivers the index

(5) $$\begin{array}{cc} i^{\min} & =\underset{i}{arg min}\left(\Delta t_{i}\right) \end{array}$$

of the shortest absolute difference $\Delta t_{i^{\min}}$. If $\Delta t_{i^{\min}}$ falls below the threshold $\theta^{\mathrm{LA}}$, the corresponding time of interest $t_{i}^{\mathrm{TI}}$ with $i=i^{\min}$ is a swallow onset. The difference between the time of interest with index $i^{\min}$ and the reference time with index *r* defines the detection delay

(6) $$\begin{array}{c} d=t_{i^{\min}}^{\mathrm{TI}}-t_{r}^{\mathrm{RT}}. \end{array}$$

The time $t_{i^{\min}}^{\mathrm{TI}}$ is removed from the series to prevent multiple assignments. Iterating over all reference times $t_{r}^{\mathrm{RT}}$ with r=1,2,…,R defines $I^{t}$ correctly detected swallow onsets and produces a vector of detection delays d containing $I^{t}$ detection delays. The remaining $I^{f}$ times of interest count as non-swallow events.

### 2.3. Evaluation Scores

An objective analysis of the timing and performance of swallow onset detection requires the definition of evaluation scores.

#### 2.3.1. Timing

Evaluating the timing of swallow onset detection employs the mean

(7) $$\begin{array}{c} \mu^{d}=\frac{1}{I^{t}}\sum_{i=1}^{I^{t}}d_{i} \end{array}$$

and standard deviation

(8) $$\begin{array}{c} \sigma^{d}=\sqrt{\frac{1}{I^{t}}\sum_{i=1}^{I^{t}}{\left(d_{i}-\mu^{d}\right)}^{2}} \end{array}$$

of the detection delays $d=[d_{1}\;d_{2}\;\ldots d_{i}\;\ldots \;d_{I^{t}}]$, for the $I^{t}$ detected swallow onset. The mean represents the average delay and the standard deviation measures the scattering of the detected swallow onsets relative to the manually marked references. Therefore, a small mean and standard deviation of the detection delays represent a precise timing of swallow onset detection.

#### 2.3.2. Preselection

The BI/EMG-based DoSO employs a preselection of potential swallow onsets in the BI data. The classifier uses a feature vector extracted from the BI and EMG data before the times of potential swallow onsets to discriminate between swallow onsets and non-swallow events. The ratio

(9) $$\begin{array}{cc} \Upsilon & =\frac{I^{t}}{I^{f}} \end{array}$$

of swallow onsets $I^{t}$ to non-swallow events $I^{f}$ is essential for classifier training. The optimal ratio Υ is close to one because the training works best with balanced data sets. The preselection’s sensitivity $S^{\mathrm{PS}}$ measures the share of detected swallow onsets from the number of swallow onsets in the data given as reference times $t_{r}^{\mathrm{RT}}$, which correspond to the times of manual annotations. Calculating the sensitivity of the preselection

(10) $$\begin{array}{cc} S^{\mathrm{PS}} & =\frac{I^{t}}{R} \end{array}$$

employs the number of identified swallow onsets $I^{t}$ and the number of reference times *R*.

#### 2.3.3. Detection Performance

Evaluating the performance of swallow onset detection uses standard scores for classifier evaluation (see, e.g., [42]), which employ the True Positive (TP), False Negative (FN), False Positive (FP), and True Negative (TN) classified swallow onsets to calculate the sensitivity

(11) $$\begin{array}{c} S=\frac{\mathrm{TP}}{\mathrm{TP}+\mathrm{FN}}, \end{array}$$

the precision

(12) $$\begin{array}{c} P=\frac{\mathrm{TP}}{\mathrm{TP}+\mathrm{FP}}, \end{array}$$

and the specificity

(13) $$\begin{array}{c} C=\frac{\mathrm{TN}}{\mathrm{TN}+\mathrm{FP}}. \end{array}$$

Controlling biofeedback and functional electrical stimulation demands the start of an intervention for every swallow onset represented by the sensitivity and a low number of falsely triggered interventions measured by the precision. The $F_{1}$ score unites properties of the sensitivity and precision without taking the true negative samples into account. Therefore, the $F_{1}$ score

(14) $$\begin{array}{c} F=\frac{2\mathrm{TP}}{2\mathrm{TP}+\mathrm{FP}+\mathrm{FN}}=\frac{2}{\frac{1}{P}+\frac{1}{S}} \end{array}$$

is the preferred measure for classification performance in this work. The $F_{1}$ score is the harmonic mean of the precision P and sensitivity S, representing the overall classification performance without considering the true negatives.

The evaluation of the EMG-based and BI/EMG-based DoSO employs a Leave-One-Subject-Out (LOSO) cross-validation (see Section 2.6.6), which generates a score for each subject in the test data. The median of the scores represents the average, and the interquartile range measures the dispersion of the scores. The formulation F = 0.638[0.153] stands for a median $F_{1}$ score of 0.638 with an interquartile range of 0.153.

### 2.4. Preprocessing of BI and EMG Data

The bioimpedance data change slowly and contain additive noise. Removing the noise through a third-order Butterworth low-pass filter with a 15 Hz cut-off frequency provides a maximum group delay of 0.03 s. Downsampling of the low-pass-filtered BI signal from 4000 Hz to 100 Hz reduces the computational expense of the BI-based preselection of swallow onsets.

After eliminating spikes, the EMG preprocessing removes the offset using a third-order high-pass filter with a 30 Hz cut-off frequency. Three notch filters with center frequencies of 50 Hz, 150 Hz, and 150 Hz suppresses power line disturbances in the EMG data. A third-order high-pass filter with a 30 Hz cut-off frequency and a second-order low-pass filter with a 300 Hz cut-off attenuate the transfer function of a whitening filter in the low- and high-frequency ranges. This whitening filter reverts the transfer function of the adhesive EMG electrodes and removes disturbances in a frequency range above 300 Hz.

In the following sections, ${\mathrm{EMG}}^{\mathrm{raw}}$ refers to the raw EMG data and EMG to the cleaned (preprocessed) EMG data. The feature calculation additionally utilizes the low-frequency trend in the electromyography tEMG of the raw EMG data generated by a third-order Butterworth low-pass filter with a 10 Hz cut-off frequency applied to the raw EMG data.

Most filter parameters were selected by visual inspection of the filter results on exemplary data. The aim was to find a trade-off between the desired filter effect and phase delays. The whitening filter and its parameters were taken from [10].

### 2.5. EMG-Based Detection of Swallow Onsets

EMG-based detection of swallow onsets employs a threshold method to detect onsets of active EMG. The method utilizes the samples of the EMG envelope in a sliding window, adopting the proposal by Li et al. [23].

#### 2.5.1. Detection of Active EMG Onsets

After preprocessing of the raw EMG data, the sample rate is reduced from 4000 Hz to 1000 Hz. The estimate of the standard deviation $\sigma^{0}$ at rest utilizes a sliding window approach on the cleaned EMG data. The data window includes $N_{\sigma }=250$ samples and has a shift of one sample. The online estimate of $\sigma^{0}$ is updated every time the standard deviation in the EMG data window falls below the current value of $\sigma^{0}$. Thus, the estimated standard deviation $\sigma^{0}$ at rest converges to the minimum in the measurement, which might cause inaccurate detection at the measurement’s beginning and reduce the performance of the EMG-based detection. A third-order low-pass filter with a 10 Hz cut-off frequency smooths the rectified clean EMG data and yields the EMG envelope signal ${\mathrm{eEMG}}_{m}$.

Two parameters control the threshold procedure: the window length *w* and the threshold factor $\theta^{0}$. The threshold

(15) $$\begin{array}{c} \theta^{\mathrm{EMG}}=\theta^{0}\;\cdot \;\sigma^{0} \end{array}$$

to detect active EMG onsets is the product of the threshold factor $\theta^{0}\in R^{+}$ and the current standard deviation at rest $\sigma^{0}$. Detecting an active EMG onset at sample index $n^{\mathrm{ON}}$ requires all samples in a window with *w* samples before the onset sample ${\mathrm{eEMG}}_{n^{\mathrm{ON}}}$ to exceed the threshold $\theta^{\mathrm{EMG}}$

(16) $$\begin{array}{c} {\mathrm{eEMG}}_{m}>\theta^{\mathrm{EMG}}\;\;\mathrm{with}\;\;m=n^{\mathrm{ON}}-w+1,\ldots ,n^{\mathrm{ON}}. \end{array}$$

**Figure 3:**
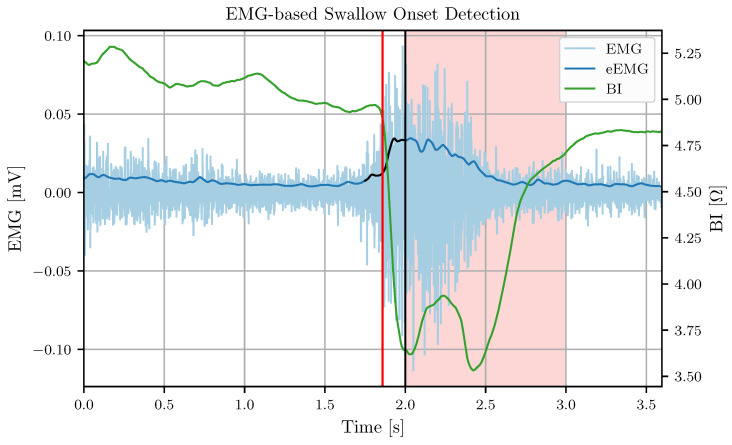
A visualization of the cleaned EMG (EMG), the envelope EMG (eEMG), and the BI of a single swallow for EMG-based swallow onset detection. The black section of the eEMG trace highlights the *w* samples of eEMG that exceed $\theta^{\mathrm{EMG}}$. The red vertical line denotes the manually marked swallow onset, while the black vertical line denotes the time of the detected swallow onset. The light red area marks the period with disabled onset detection that starts at a detected onset.

After a detected EMG onset at index $n^{\mathrm{ON}}$, EMG onset detection is disabled for at least 1.0 s and until a sample ${\mathrm{eEMG}}_{i}$ with $i>n^{\mathrm{ON}}$ falls below the threshold $\theta^{\mathrm{EMG}}$. During this period, the biofeedback or FES intervention will take place so a further triggering is not meaningful anyway. Figure 3 illustrates the threshold procedure for active EMG onset detection and the eEMG data of a single swallow.

#### 2.5.2. Label Assignment

The evaluation of the EMG-based DoSO requires the assignment of the $I^{\mathrm{ON}}$ onsets of active EMG at the times

(17) $$\begin{array}{c} t_{i}^{\mathrm{ON}}=\frac{n_{i}^{\mathrm{ON}}}{f_{T}}\;\mathrm{with}\;i=1,2,\ldots ,I^{\mathrm{ON}} \end{array}$$

to the *R* reference times of swallow onsets

(18) $$\begin{array}{c} t_{r}^{\mathrm{RT}}\;\mathrm{with}\;r=1,2,\ldots ,R, \end{array}$$

marked by the expert. Assigning the times of active EMG onsets $t_{i}^{\mathrm{ON}}$ to the reference times $t_{r}^{\mathrm{RT}}$ employs the assignment procedure of Section 2.2 with a threshold of $\theta^{\mathrm{LA}}=0.5s$ (for justification of this choice, see Section 2.6.2). The assignment procedure creates $I^{t}$ swallow onsets counting as a true positive and $I^{f}$ onsets of non-swallow events rated as a false positive. After finishing the *R* iterations of the assignment procedure, the unassigned reference times $t_{r}^{\mathrm{RT}}$ count as false negatives. The number of true negatives

(19) $$\begin{array}{c} \mathrm{TN}=M-N_{\mathrm{skip}}-N_{\mathrm{start}}-\mathrm{FN}-I^{f}-I^{t} \end{array}$$

equals the sample number *M* of the downsampled EMG data vector minus the excluded samples $N_{\mathrm{skip}}$, the false EMG onsets $I^{f}$, the false negatives FN, the true EMG onsets $I^{t}$, the skipped samples $N_{\mathrm{skip}}$, and excluded samples $N_{\mathrm{start}}$ at the start of an EMG measurement defined by the maximum of *w* or $N_{\sigma }$. Finally, the confusion matrix enables the calculation of evaluation scores, such as sensitivity, precision, and $F_{1}$ score for the subjects.

#### 2.5.3. Optimization and Evaluation

The optimization of the parameters $\theta^{0}$ and *w* employs a grid search for testing the parameter pairs ($\theta_{k}^{0}$, $w_{l}$) generated by combining $L_{\theta }$ values of the threshold factor

(20) $$\begin{array}{cc} \theta^{0} & =[1\;1.5\;2\;2.5\;3\;3.5\;4\;4.5\;5\;5.5\;6.0\;6.5\;7.0] \end{array}$$

and $L_{w}$ realizations of the window length

(21) $$\begin{array}{cc} w & =[50\;75\;100\;125\;150\;175\;200\;225\;250\;275\;300]. \end{array}$$

The grid search produces two matrices: $F\in R^{L_{\theta }\times L_{w}}$ and $\mu \in R^{L_{\theta }\times L_{w}}$, containing the $F_{1}$ score and mean detection delay for each parameter pair ($\theta_{k}^{0}$, $w_{l}$), respectively. Selecting the optimal parameter pair (${\hat{\theta }}^{0}$, $\hat{w}$) from the matrices applies two conditions. First, determining the indices

(22) $$\begin{array}{c} \left(k,l\right)=\underset{\mu \left(k,l\right)<\theta^{\mu^{d}}}{\arg}\left(\mu \right) \end{array}$$

of the elements in the mean detection delay matrix μ that fall below the threshold $\theta^{\mu^{d}}$ to limit the mean detection delay of the EMG-based DoSO. The selection of the threshold $\theta^{\mu^{d}}$ will be discussed later on in Section 3.2.1. Second, the element $F_{\hat{k},\hat{l}}$ of the $F_{1}$ score matrix **F**

(23) $$\begin{array}{c} \left(\hat{k},\hat{l}\right)=\underset{k\in k,l\in l}{arg max}F_{k,l} \end{array}$$

with the highest $F_{1}$ score among the elements (k,l) defines the optimal parameter pair (${\hat{\theta }}^{0}=\theta_{\hat{k}}^{0}$, $\hat{w}=w_{\hat{l}}$) for the given data.

The LOSO cross-validation iteratively employs the measurements of all subjects except for subject s∈{1,2,…,S} to determine the optimal parameter pair (${\hat{\theta }}^{0}$, $\hat{w}$) for subject *s* with a grid search. Detecting the active EMG onsets in the EMG measurements of subject *s* with the optimized parameter pair (${\hat{\theta }}^{0}$, $\hat{w}$) yields the evaluation score and the detection delay for the subject *s*. The final score is the median and interquartile range of the *S* evaluation scores produced by the LOSO-cross-validation iterations. Computing the mean and standard deviation of the detection delays of all *S* subjects completes the analysis.

### 2.6. BI/EMG-Based Detection of Swallow Onsets

BI/EMG-based detection of swallow onsets involves of two stages. First, a BI-based preselection determines times of potential swallow onsets. Second, a classifier uses feature vectors extracted from BI and EMG data before the times of potential swallow onsets to distinguish swallow onsets and non-swallow events. Training, hyperparameter optimization, and evaluation of a classifier require a set of labeled features. This section describes the BI-based preselection, the feature extraction, the feature optimization, the hyperparameter optimization, and the evaluation procedure.

#### 2.6.1. BI-Based Preselection

Algorithm 1 presents the BI-based preselection that searches for a significant signal drop after a detected maxima. The function Initialization$\left({\mathrm{BI}}_{1}\right)$ sets **max** to False, and ${\mathrm{BI}}_{\max}$, ${\mathrm{BI}}_{m-1}$, and ${\mathrm{BI}}_{m-2}$ to the first sample ${\mathrm{BI}}_{1}$ of the low-pass-filtered BI data. Calling the preselection procedure Preselection$\left({\mathrm{BI}}_{m}\right)$ with the subsequent samples ${\mathrm{BI}}_{m}$ of the BI data returns True for a potential swallow onset with the index *m*. The detection of local maxima compares three subsequent samples of the BI data vector stored in the variables ${\mathrm{BI}}_{m}$, ${\mathrm{BI}}_{m-1}$, and ${\mathrm{BI}}_{m-2}$. If ${\mathrm{BI}}_{m-1}$ exceeds ${\mathrm{BI}}_{m}$ and ${\mathrm{BI}}_{m-2}$, the data sample ${\mathrm{BI}}_{m-1}$ represents a new local maximum. ${\mathrm{BI}}_{\max}$ saves the value of the new local maximum ${\mathrm{BI}}_{m-1}$, and the flag **max** becomes True to enable the search for the BI drop.
**Algorithm 1** BI-based Preselection1:**procedure** Initialization(${\mathrm{BI}}_{1}$)
2:    ${\mathrm{BI}}_{\max}\leftarrow {\mathrm{BI}}_{1}$▹ current maximum3:    ${\mathrm{BI}}_{m-1}\leftarrow {\mathrm{BI}}_{1}$▹ previous sample4:    ${\mathrm{BI}}_{m-2}\leftarrow {\mathrm{BI}}_{1}$▹ pre-previous sample5:    max←False▹ flag for maximum search6:**end procedure**7:8:**procedure** Preselection(${\mathrm{BI}}_{m}$)9:    out ← False10:11:    **if** ${\mathrm{BI}}_{m-2}<{\mathrm{BI}}_{m-1}$ **and** ${\mathrm{BI}}_{m-1}>{\mathrm{BI}}_{m}$ **then**▹ reset local maximum search12:        ${\mathrm{BI}}_{\max}\leftarrow {\mathrm{BI}}_{m-1}$13:        max← True14:    **end if**15:16:    **if** ${\mathrm{BI}}_{\max}-{\mathrm{BI}}_{m}>\theta^{\mathrm{PS}}$ **and** max = True **then**▹ check threshold17:        max← False18:        out← True19:    **end if**20:21:    ${\mathrm{BI}}_{m-2}\leftarrow {\mathrm{BI}}_{m-1}$▹ save values for local maximum search22:    ${\mathrm{BI}}_{m-1}\leftarrow {\mathrm{BI}}_{m}$23:24:    **return** out25:**end procedure**

Comparing the BI difference (${\mathrm{BI}}_{\max}-{\mathrm{BI}}_{m}$) between the current local maximum ${\mathrm{BI}}_{\max}$ and the actual BI sample ${\mathrm{BI}}_{m}$ to the threshold $\theta^{\mathrm{PS}}$ determines potential swallow onsets. If the BI difference exceeds the threshold $\theta^{\mathrm{PS}}$, **max** becomes False, and the return value **out** is True. Setting the flag **max** = False prevents detecting multiple potential swallow onsets after a local maximum. A new local maximum reactivates the search for potential swallow onsets because **max** becomes True.

#### 2.6.2. Label Assignment

Applying preselection to a BI data vector BI yields times of interest $t_{i}^{\mathrm{TI}}=m_{i}\;\cdot \;\frac{1}{f_{T}}$ for the indices $m_{i}$ of the BI samples ${\mathrm{BI}}_{m_{i}}$ if the function $Preselection({\mathrm{BI}}_{m})$ returns True. The times of interest $t_{i}^{\mathrm{TI}}$ with i=1,2,…,I represent potential swallow onsets, consisting of swallow onsets and non-swallow events. Creating a data set $D=\{\left(y_{i},x_{i},s_{i}\right)\}$ of samples containing feature vectors $x_{i}\in R^{J}$, subject indices $s_{i}\in \left\{0,1,\ldots ,S\right\}$, and class labels $y_{i}\in \left\{0,1\right\}$ requires the assignment of the class labels. Assigning the class labels employs the procedure presented in Section 2.2.

Setting the threshold interval to $\theta^{\mathrm{LA}}\;=\;0.5\;s$ considers the mean 0.39 s and standard deviation 0.14 s of the duration $t_{\min}$ from the start to the minimum of the BI valleys, according to Schultheiss et al. [43]. The assignment procedure generates $I^{t}$ swallow onset samples (true samples), $I^{f}$ non-swallow event samples (false samples), and a vector of detection delays d with an entry for each swallow onset.

In some cases, preselection detects multiple potential swallow onsets on the decreasing edge of a BI swallow valley. The assignment of class label marks one of the potential swallow onsets as a swallow onset and the remaining potential swallow onsets as non-swallow events, even if the times $t_{i}^{\mathrm{TI}}$ fall on a decreasing edge of a BI swallow valley. Thus, the corresponding feature vectors have the properties of swallow onsets labeled as non-swallow events, which impairs the classifier training.

Furthermore, the intended use of BI/EMG-based DoSO is to trigger FES or biofeedback. Therefore, removing non-swallow events falling in the interval $[t_{i}^{T},\;t_{i}^{T}+\theta^{\mathrm{skip}}[$ with length $\theta^{\mathrm{skip}}$ after a swallow onset at $t_{i}^{T}$ from D respects the use case of the BI/EMG-based DoSO because triggering biofeedback or FES during an active intervention is pointless. The interval length of $\theta^{\mathrm{skip}}$ = 1.0 s respects the 0.763 s mean and 0.205 s standard deviation of the BI swallow valley duration $t_{\mathrm{end}}$ presented by Schultheiss et al. [43].

#### 2.6.3. Feature Extraction

**Figure 4:**
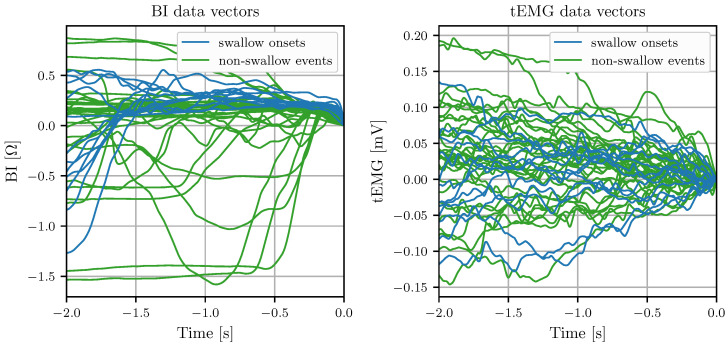
**Left:** Visualization of the BI data vectors of swallow onsets (12) and non-swallow events (34) in a healthy subject, shifted to zero at the time zero of potential swallow onsets. **Right:** Visualization of the tEMG data vectors of swallow onsets and non-swallows shifted to zero at the time zero of potential swallow onsets.

**Figure 5:**
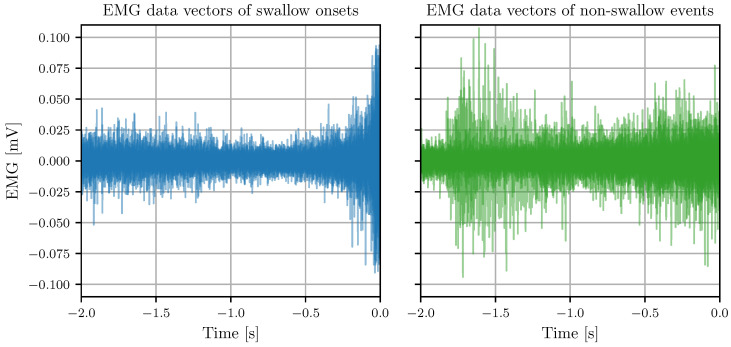
A visualization of EMG data vectors of swallow onsets and non-swallow events in a healthy subject preceding the potential swallow onsets at time zero. The left subplot shows the EMG vectors of 12 swallow onsets (blue), and the right subplot shows the EMG vectors of 34 non-swallow events (green).

Features are extracted from the BI, EMG, and tEMG signals for time windows preceding any potential swallow onset detected by the BI-based preselection. Figure 4 and Figure 5 show examples of the signals for swallow onsets and non-swallow events in a healthy subject.

For the calculation of the *j*-th element of the feature vector $x_{i}$ from a signal *Z* (BI, EMG, or tEMG), a data vector is generated

(24) $$\begin{array}{cc} \; & Z_{i,j}=\left[Z_{m_{i}-M_{j}+1}\;\ldots \;Z_{m_{i}-1}\;Z_{m_{i}}\right] \end{array}$$

using $M_{j}$ samples up to the sample index $m_{i}$ indicating a potential swallow onset.

Maximizing the feature relevance yields an optimal sample number $M_{j}^{\mathrm{opt}}$ for the data vector of a feature. The feature vectors $x_{i}$ consist of *J* different features $x_{i,j}$. Table A1 presents a complete list of all features with the corresponding index *j*.

The standard deviation measures the scatter of the data vectors. Calculating the standard deviation $\sigma_{i}^{\mathrm{BI}}$ of BI data vectors ${\mathrm{BI}}_{i}$, and the standard deviation $\sigma_{i}^{\mathrm{tEMG}}$ of EMG data vectors ${\mathrm{EMG}}_{i}$ provide features concerning the data scatter.

(25) $$\begin{array}{cc} x_{i,1} & =\sigma_{i}^{\mathrm{tEMG}}=\sigma \left({\mathrm{tEMG}}_{i,1}\right) \end{array}$$

(26) $$\begin{array}{cc} x_{i,4} & =\sigma_{i}^{\mathrm{BI}}=\sigma \left({\mathrm{BI}}_{i,4}\right) \end{array}$$

The features ${♈}_{i}^{\mathrm{BI}}$ and ${♈}_{i}^{\mathrm{tEMG}}$ represent the number of samples in the data vectors ${\mathrm{BI}}_{i,j}$ and ${\mathrm{tEMG}}_{i,j}$ exceeding the reference values ${\mathrm{tEMG}}_{m_{i}}$ and ${\mathrm{BI}}_{m_{i}}$, respectively, divided by the sample number of the data vectors.

(27) $$\begin{array}{cc} x_{i,2} & ={♈}_{i}^{\mathrm{tEMG}}=\frac{\left|\{{\mathrm{tEMG}}_{i,2}|{\mathrm{tEMG}}_{l}>{\mathrm{tEMG}}_{m_{i}},l=m_{i}-M_{2}+1\;\ldots ,m_{i}-1\}\right|}{M_{2}} \end{array}$$

(28) $$\begin{array}{cc} x_{i,5} & ={♈}_{i}^{\mathrm{BI}}=\frac{\left|\{{\mathrm{BI}}_{i,5}|{\mathrm{BI}}_{l}>{\mathrm{BI}}_{m_{i}},l=m_{i}-M_{5}+1\;\ldots ,m_{i}-1\}\}\right|}{M_{5}} \end{array}$$

Here, |{·}| represents the cardinality of a set.

The next feature $x_{i,9}$ is the Average Amplitude Change (AAC) of the EMG data vectors ${\mathrm{EMG}}_{i,9}$. This feature estimates the extent of EMG activation.

(29) $$\begin{array}{cc} x_{i,9} & ={\mathrm{AAC}}_{i}=\frac{1}{M_{9}}\sum_{l=m_{i}-M_{9}+1}^{m_{i}-1}\left|{\mathrm{EMG}}_{l+1}-{\mathrm{EMG}}_{l}\right| \end{array}$$

Furthermore, a temporal analysis of BI, EMG, and tEMG data extracts essential information. The method divides the $M_{j}$ data samples into $W_{j}\geq 2\in N$ windows containing $\Delta_{j}\in N$ samples. The sample number $M_{j}$ in a data vector $Z_{i,j}$ is restricted to multiples of the window length $\Delta_{j}$. The mean

(30) $$\begin{array}{cc} \mu_{n}\left(Z_{i,j}\right) & =\frac{1}{\Delta_{j}}\sum_{l=\left(n-1\right)\;\cdot \;\Delta_{j}+1}^{n\;\cdot \;\Delta_{j}}Z_{l+m_{i}-M_{j}} \end{array}$$

and standard deviation

(31) $$\begin{array}{cc} \sigma_{n}\left(Z_{i,j}\right) & =\sqrt{\frac{1}{\Delta_{j}}\sum_{l=\left(n-1\right)\;\cdot \;\Delta_{j}+1}^{n\;\cdot \;\Delta_{j}}{\left(Z_{l+m_{i}-M_{j}}-\mu_{n}\left(Z_{i,j}\right)\right)}^{2}} \end{array}$$

of $n=1,2,\ldots ,W_{j}$ windows are the basis of the temporal-structure features.

Two EMG features utilize the standard deviations $\sigma_{n}\left(z\right)$, the index of the window with maximal standard deviation ${\bar{\sigma }}_{i}^{\mathrm{EMG}}$, and the index of the window with minimal standard deviation ${\underline{\sigma }}_{i}^{\mathrm{EMG}}$.

(32) $$\begin{array}{cc} x_{i,11} & ={\bar{\sigma }}_{i}^{\mathrm{EMG}}=\underset{n}{arg max}\left(\sigma_{n}\left({\mathrm{EMG}}_{i,11}\right)\right) \end{array}$$

(33) $$\begin{array}{cc} x_{i,12} & ={\underline{\sigma }}_{i}^{\mathrm{EMG}}=\underset{n}{arg min}\left(\sigma_{n}\left({\mathrm{EMG}}_{i,12}\right)\right) \end{array}$$

Additionally, the difference

(34) $$\begin{array}{cc} x_{i,10} & =\delta_{i}^{\mathrm{EMG}}=\sigma_{2}\left({\mathrm{EMG}}_{i,10}\right)-\sigma_{1}\left({\mathrm{EMG}}_{i,10}\right) \end{array}$$

between the standard deviations of two subsequent windows with $W_{10}=2$ before the times of interest $t_{i}^{\mathrm{TI}}$ models the steepness of the active EMG onset.

The same method assesses the time structure of ${\mathrm{BI}}_{i,j}$ and ${\mathrm{tEMG}}_{i,j}$ data vectors. The value of the highest standard deviation ${\hat{\sigma }}_{i}^{\mathrm{BI}}$, the index of the highest standard deviation ${\bar{\sigma }}_{i}^{\mathrm{BI}}$, and the index of the lowest standard deviation ${\underline{\sigma }}_{i}^{\mathrm{BI}}$ among $W_{j}$ windows of the BI data vectors ${\mathrm{BI}}_{i,j}$ capture information about prominent BI alterations. The value of the highest standard deviation ${\hat{\sigma }}_{i}^{\mathrm{tEMG}}$ among $W_{j}$ windows of the tEMG data vectors ${\mathrm{tEMG}}_{i,j}$ extends the feature set.

(35) $$\begin{array}{cc} x_{i,6} & ={\hat{\sigma }}_{i}^{\mathrm{BI}}=\max_{n}\left(\sigma_{n}\left({\mathrm{BI}}_{i,6}\right)\right) \end{array}$$

(36) $$\begin{array}{cc} x_{i,7} & ={\bar{\sigma }}_{i}^{\mathrm{BI}}=\underset{n}{arg max}\left(\sigma_{n}\left({\mathrm{BI}}_{i,7}\right)\right) \end{array}$$

(37) $$\begin{array}{cc} x_{i,8} & ={\underline{\sigma }}_{i}^{\mathrm{BI}}=\underset{n}{arg min}\left(\sigma_{n}\left({\mathrm{BI}}_{i,8}\right)\right) \end{array}$$

(38) $$\begin{array}{cc} x_{i,3} & ={\hat{\sigma }}_{i}^{\mathrm{tEMG}}=\max_{n}\left(\sigma_{n}\left({\mathrm{tEMG}}_{i,3}\right)\right) \end{array}$$

#### 2.6.4. Feature Optimization

Radivojac et al. [44] defines feature relevance as the difference between the probability distributions $f_{A}\left(x_{i,j}\right)$ and $f_{B}\left(x_{i,j}\right)$ of the true $A=\{D|y_{i}=1\}$ and false $B=\{D|y_{i}=0\}$ samples of a feature in a data set $D=\{\left(y_{i},x_{i},s_{i}\right)\}$ with i=1,2,…,I samples. The overlap measures the similarity of $f_{A}\left(x_{i,j}\right)$ and $f_{B}\left(x_{i,j}\right)$ without further restraints on the distributions [45]. Usually, a Kernel Density Estimation (KDE) generates approximations ${\hat{f}}_{A}\left(x_{i,j}\right)$ and ${\hat{f}}_{B}\left(x_{i,j}\right)$ of the probability distributions $f_{A}\left(x_{i,j}\right)$ and $f_{B}\left(x_{i,j}\right)$ based on the sets *A* and *B*. Integrating over the minimum of approximated probability distributions ${\hat{f}}_{A}\left(x_{i,j}\right)$ and ${\hat{f}}_{B}\left(x_{i,j}\right)$ yields an estimation of the overlap

(39) $$\begin{array}{cc} \eta (A,B) & =\int_{-\infty }^{\infty }\min\left[{\hat{f}}_{A}\left(x_{i,j}\right),{\hat{f}}_{B}\left(x_{i,j}\right)\right]\;dx_{i,j}. \end{array}$$

In this work, the feature optimization employs a custom method to estimate the overlap (see Appendix A).

The feature optimization for the BI/EMG-based DoSO uses the relevance measure (39) to optimize the sample number $M_{j}\in N^{+}$ of the data vectors for feature extraction. Calculating feature values $x_{i,j}$ for a series of increasing sample numbers $M_{j}=\Delta_{j}n$ with $n=2,\ldots ,N_{j}$ yields a series of overlap values $\eta (A\left(M_{j}\left(n\right)\right),B\left(M_{j}\left(n\right)\right))$. Here, $\Delta_{j}$ and $N_{j}$ are positive integers defined for each feature in Table A1. Therefore, the optimal sample number $M_{j}^{\mathrm{opt}}=\Delta_{j}n_{j}^{\mathrm{opt}}$ is the sample number with the index of smallest overlap.

(40) $$\begin{array}{cc} n_{j}^{\mathrm{opt}} & =\underset{n=2,\ldots ,N_{j}}{arg min}\left(\eta \left(A\left(M_{j}\left(n\right)\right),B\left(M_{j}\left(n\right)\right)\right)\right) \end{array}$$

Estimating the optimal sample number utilizes the data of data series I, II, III, and IV. The feature extraction employs the optimized sample numbers $M_{j}^{\mathrm{opt}}$ of the data vectors to create the data set for hyperparameter optimization and evaluation. Finally, $W_{j}=n_{j}^{\mathrm{opt}}$ is used for the features 3, 6, 7, 8, 11, and 12.

**Figure 6:**
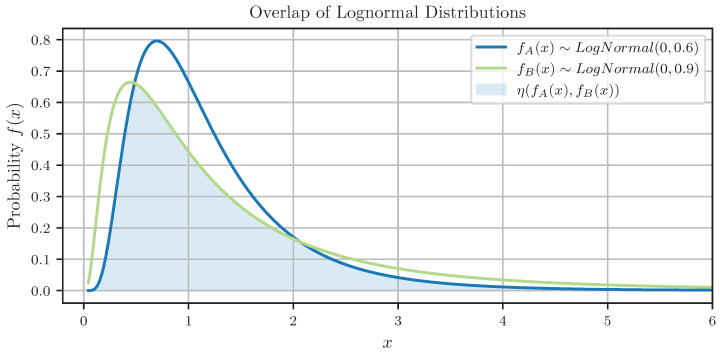
Illustration of the overlap η(A,B) between two Log-normal probability-density functions $f_{B}\left(x\right)∼LogNormal\left(0,0.6\right)$ and $f_{A}\left(x\right)∼LogNormal\left(0,0.9\right)$.

Figure 6 presents a visualization of the overlap between two Log-normal probability-density functions $f_{B}\left(x\right)∼LogNormal\left(0,0.6\right)$ and $f_{A}\left(x\right)∼LogNormal\left(0,0.9\right)$ and clarifies the capability of the overlap to measure the similarity between two probability distributions.

#### 2.6.5. Hyperparameter Optimization

Choosing a classifier involved a comparison of Random Forest (RF), support vector machines, k-nearest neighbors, and multi-layer perceptron classifiers that included five methods for data scaling. The RF classifier achieved the best results for data of dysphagia patients and is independent of the scaling method. Therefore, this work employs the RF classifier and a standard scaler to distinguish swallow onsets from non-swallow events.

Usually, classifiers provide a set of *P* hyperparameters controlling the training process. Determining the optimal hyperparameter vector $\lambda^{\mathrm{opt}}$ requires the evaluation of multiple realizations of hyperparameter vectors

(41) $$\begin{array}{c} \lambda_{l}=\left[\begin{array}{c} \lambda_{l,1}\;\lambda_{l,2}\;\ldots \;\lambda_{l,P} \end{array}\right] \end{array}$$

with l=1,2,…,L for given hyperparameter ranges.

Grid search is a straightforward approach for hyperparameter optimization that reduces the search space $\Lambda =\{\lambda_{1},\lambda_{2},\ldots ,\lambda_{l},\ldots ,\lambda_{L}\}$ to L∈N equal distant points, given by predefined hyperparameter vectors $\lambda_{l}$ generated from predefined hyperparameter ranges.

Random search generates the hyperparameter vectors $\lambda_{l}$ by sampling the hyperparameter values $\lambda_{l,p}$ randomly from the predefined ranges. Bergstra et al. [46] argue that random search outperforms grid search in higher dimensional search spaces.

Hyperparameter optimization evaluates the hyperparameter response function Ψ for each $\lambda_{l}$ to determine the optimal hyperparameter vector

(42) $$\begin{array}{c} \lambda^{\mathrm{opt}}=\underset{\lambda \in \Lambda }{arg max}\left(\Psi \right). \end{array}$$

A mixed hyperparameter optimization is applied in this work. Table 2 presents all optimized hyperparameters. Grid search optimizes the class weighting, and random search searches four additional parameters.

The applied Python library Sklearn [47] allows the definition of weights $w_{0}$ and $w_{1}$ for the two classes (0—non-swallows and 1—swallow onsets):

(43) $$\begin{array}{c} \omega_{0}=\frac{{\mathrm{weight}}_{0}\;\cdot \;\left|\left\{D|y_{i}=0\right\}\right|}{2\;\cdot \;|D|}=\frac{{\mathrm{weight}}_{0}\;\cdot \;I^{f}}{2I}, \end{array}$$

(44) $$\begin{array}{c} \omega_{1}=\frac{{\mathrm{weight}}_{1}\;\cdot \;\left|\left\{D|y_{i}=1\right\}\right|}{2|D|}=\frac{{\mathrm{weight}}_{1}\;\cdot \;I^{t}}{2I}. \end{array}$$

The grid search is used for the hyperparameter **weight**_1_ by evaluating $L_{1}=6$ values

(45) $$\begin{array}{c} {\mathrm{weight}}_{1}\in \left\{0.5,1.0,1.5,2.0,2.5,3.0\right\} \end{array}$$

with ${\mathrm{weight}}_{0}=1$.

The hyperparameters with random search are **ccp_alpha** for cost-complexity pruning, **min_impurity_decrease** and **min_weight_fraction_leaf** for early stopping during the tree construction, and **max_samples** to test different sizes of the bagging data sets. The number of evaluated vectors for these four parameters is $L_{2}=200$.

Setting the hyperparameters **min_samples_split** = 2, **min_samples_leaf** = 1, **max_dept** = None, and **max_leaf_nodes** = None disables these mechanisms.

The remaining hyperparameters **n_estimators** = 100, **criterion** = gini, **max_features** = sqrt, **bootstrap** = True, **random_state** = 1, and **class_weight** = balanced remain in the default state.

#### 2.6.6. Classifier Selection and Test

Classifier selection and unbiased testing with respect to hyperparameter optimization requires a nested LOSO cross-validation. The outer LOSO cross-validation iteratively splits the complete data D into evaluation data $D^{e}=\left\{D|s_{i}\neq s^{\mathrm{test}}\right\}$ and test data $D^{\mathrm{test}}=\left\{D|s_{i}=s^{\mathrm{test}}\right\}$ concerning a test subject $s^{\mathrm{test}}=1,2,\ldots ,S$.

The inner LOSO cross-validation trains and evaluates the classifier for all $L=L_{1}\;\cdot \;L_{2}$ hyperparameter vectors on the data $D^{e}$ of the remaining subjects. Iterating over all (S−1) remaining subjects and hyperparameter vectors $\lambda_{l}$ with l=1,2,…,L in the inner LOSO cross-validation generates a matrix $O_{s^{\mathrm{test}}}\in R^{L\times (S-1)}$ of F_1_ scores and a matrix of complexity measures $C_{s^{\mathrm{test}}}\in R^{L\times (S-1)}$. The latter contains the leaf numbers in the decision trees of the random forest classifier as proposed by Breiman [48].

The classifier selection and test utilize the matrices $O_{s^{\mathrm{test}}}$ and $C_{s^{\mathrm{test}}}$ to determine the corresponding index $l^{\mathrm{opt}}$ of the optimal hyperparameter vector for the classifier training. The *l*-th rows of the matrices link to the hyperparameter vector $\lambda_{l}$ and contain the obtained scores/complexity measures for the (S−1) subjects. The vectors $o_{l}$ and $c_{l}$ denote the *l*-th row of $O_{s^{\mathrm{test}}}$ and $C_{s^{\mathrm{test}}}$, respectively. The selection and test process involves the following steps [49,50]:

1. Determine the mean values $\mu_{l}$ for all rows of $O_{s^{\mathrm{test}}}$.
2. Find the index of the largest mean value: $l^{\max}=\arg_{l}\max\left(\mu_{l}\right)$.
3. Calculate the standard error $\sigma^{\mathrm{se}}=\mathrm{std}(o_{l^{\max}})/\sqrt{S-1}$ [49] and the modified standard error $\sigma_{l}^{\mathrm{mod}}=\sigma^{\mathrm{se}}\sqrt{1-\rho_{l}}$ for each row [50]. Here, $\rho_{l}$ is the correlation of the vectors $o_{l^{\max}}$ and $o_{l}$.
4. Select all rows of $O_{s^{\mathrm{test}}}$ whose $\mu_{l}$ lie in the interval $\left[\mu_{l^{\max}}-\sigma_{l}^{\mathrm{mod}},\mu_{l^{\max}}\right]$ and select the one that has the lowest mean of the corresponding rows in the matrix $C_{s^{\mathrm{test}}}$ (complexity measure). This yields the index $l^{\mathrm{opt}}$ and vector $\lambda_{l^{\mathrm{opt}}}$.
5. Test the classifier linked to $\lambda_{l^{\mathrm{opt}}}$ on $D^{\mathrm{test}}$ to yield the unbiased score $\hat{o}\left[s^{\mathrm{test}}\right]$.

In summary, the selection of the optimal RF classifier considers all models whose performance scores are within the modified standard error interval and chooses the model with the lowest complexity. Finally, after completion of the outer LOSO cross-validation, the evaluation returns the test score vector $\hat{o}\in R^{S}$. The median and interquartile range summarize the test scores from the vector $\hat{o}$ for *S* subjects.

## 3. Results

### 3.1. BI-Based Preselection of Swallow Onsets

The choice of $\theta^{\mathrm{PS}}$ is critical because this parameter affects the timing and classification performance. Triggering the interventions synchronous to swallowing requires a low mean $\mu^{d}$ and standard deviation $\sigma^{d}$ of the detection delay. Since the mean BI drop lasts $t_{\min}=0.39\;s$ according to Schultheiss et al. [43], a desired mean detection delay below $\mu^{d}\leq 0.039\;s$ results in detecting swallow onsets within the first 10.0% of the BI drop duration. The classification performance will most likely increase with a more balanced ratio Υ. Thus, the choice of $\theta^{\mathrm{PS}}$ should consider both aspects. After a manual tuning, $\theta^{\mathrm{PS}}=0.18\;\Omega$ was obtained, leading to the desired mean detection delay of $\mu^{d}=0.039\;s$ and a standard deviation of $\sigma^{d}=0.079\;s$ in average for all data series.

Table 3 presents the scores used to evaluate the BI-based preselection with $\theta^{\mathrm{PS}}=0.18\;\Omega$ separately for the data series I, II, III, and IV. The mean detection delay $\mu^{d}=0.033s$ for dysphagia patients (data series IV) falls below the desired limit of 0.039 s.

Nevertheless, the differences of $\mu^{d}$ between data series are small compared to the standard deviation of the detection delays. Thus, bolus type, swallowing style, anatomical differences, and other individual factors influence detection delay. Generally, the BI-based preselection provides an excellent sensitivity $S^{\mathrm{PS}}$ of from 0.977 to 0.998 and a sufficient ratio Υ of swallow to non-swallow events of from 0.114 to 0.256.

### 3.2. Optimized Test Results

#### 3.2.1. EMG-Based Detection of Swallow Onsets

The optimization of the parameters for the EMG-based DoSO uses the mean detection delays from Table 3 as values for the threshold $\theta^{\mu^{d}}$ in (22).

Table 4 presents the sensitivity, precision, $F_{1}$ score, and the mean $\mu^{d}$ and standard deviation $\sigma^{d}$ of the detection delays for EMG-based DoSO. The EMG-based DoSO achieves the highest $F_{1}$ score F=0.619[0.199] for data series II. The $F_{1}$ scores F=0.553[0.135] and F=0.529[0.127] for data series I and III are more than 0.05 units lower compared to those of data series II because data set I contains the highest share of movements and the SNR of the EMG measurements in data series III is the lowest of the data series of healthy subjects. The median $F_{1}$ score F=0.289[0.496] for dysphagia patients is more than 0.3 units lower compared to data sets II and III.

The median sensitivity is high for data series I and II with S = 0.84[0.131] and S = 0.759[0.235], respectively. The EMG-based DoSO has a reduced median sensitivity S=0.5[0.678] concerning dysphagia patients in data series IV.

The mean detection delays of the EMG-based DoSO are slightly shorter than the mean detection delays of BI/EMG-based DoSO, presented in Table 5. Nevertheless, $\mu^{d}$ for EMG-based and BI/EMG-based DoSO fall in a similar range.

#### 3.2.2. BI/EMG-Based Detection of Swallow Onsets

The evaluation of the random forest classifier for the BI/EMG-based detection of swallow onsets employs the data of each data series I, II, III, and IV separately. Table 5 shows the median and interquatile range of the sensitivity, precision, $F_{1}$ score, and specificity for the BI/EMG-based DoSO. The specificity is approximately 0.95 for all data series, indicating an excellent classification of non-swallow events. The BI/EMG-based DoSO reaches a $F_{1}$ scores of F=0.705[0.191] for data series I, F=0.826[0.094] for data series II, F=0.732[0.184] for data series III, and F=0.546[0.405] for data series IV. Therefore, the BI/EMG-based DoSO performs substantially better compared to the EMG-based DoSO. The $F_{1}$ score for dysphagia patients is approximately 0.2 units lower compared to healthy subjects in data series I, II, and III. The sensitivity of the BI/EMG-based DoSO is slightly lower, but the precision is higher compared to the EMG-based DoSO. The maximization of the $F_{1}$ score yields ${\mathrm{weight}}_{1}$ values between 0.5 and 2.0.

Evaluating the random forest classifier for BI/EMG-based DoSO employs two additional data combinations generated by adding data from healthy subjects to the training data and testing the classifier with the data of dysphagia patients. Table 6 presents the evaluation scores for training with data combination A={I,II,III} or B={I,II,III,IV} evaluated with data from data series IV. The $F_{1}$ score for combination **A** in row one is slightly higher, and the $F_{1}$ score for combination **B** in row two is reduced, compared to the performance for data series IV in Table 5. Thus, extending the data for classifier training with samples from healthy subjects does not substantially enhance the classification performance for dysphagia patients.

**Figure 7:**
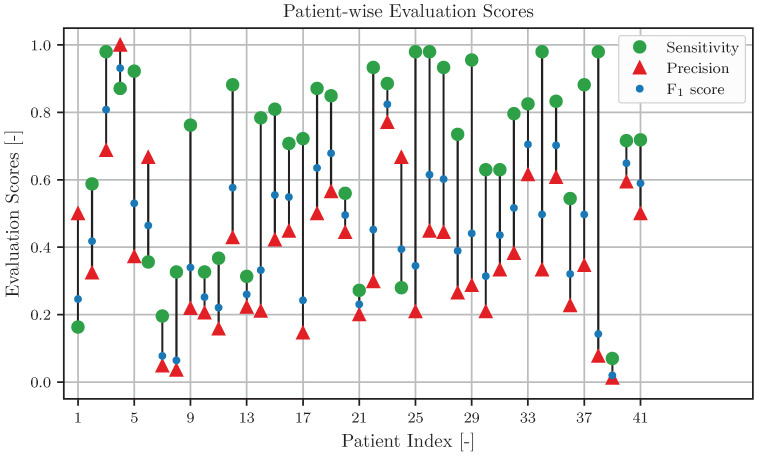
Visualization of sensitivity (green dots), precision (red triangles), and $F_{1}$ score (blue dots) of BI/EMG-based detection of swallow onsets for individual dysphagia patients. A black line connects the sensitivity and the precision of each patient to visualize the span width between the scores.

Figure 7 visualizes the sensitivity, precision, and $F_{1}$ score values of single dysphagia patients in data series IV, yielding the median and interquartile range values presented in Table 5. Usually, the sensitivity is the highest score for a patient, while the precision is lower, and the $F_{1}$ score falls between the sensitivity and precision. The sensitivity reaches a sufficient level of 0.7 for 26 out of 41 patients, resulting in a median sensitivity of S=0.871 and precision of P=0.425 for these patients. The sensitivity falls below 0.4 for ten patients, yielding a median sensitivity of S=0.297 and precision of P=0.203 for these patients. Therefore, BI/EMG-based DoSO performs well for the majority of patients but struggles for some dysphagia patients who are most likely more strongly impaired.

## 4. Discussion

The BI/EMG-based DoSO achieves considerably more precise timing compared to EMG-based DoSO. The BI-based preselection determines potential swallow onsets by detecting typical patterns in the BI measurements. Therefore, the BI-based preselection determines the timing of the BI/EMG-based DoSO. Evaluating the timing employs the mean $\mu^{d}$ and standard derivation $\sigma^{d}$ of the detection delays. The BI-based preselection achieves $\mu^{d}\left(\sigma^{d}\right)=0.039\;\left(0.079\right)\;s$ for the complete data and $\mu^{d}\left(\sigma^{d}\right)=0.033\;\left(0.1\right)\;s$ for dysphagia patients.

In contrast, the EMG-based DoSO reached a detection delay of $\mu^{d}\left(\sigma^{d}\right)=0.027\;\left(0.183\right)\;s$ for healthy subjects and $\mu^{d}\left(\sigma^{d}\right)=0.018\;\left(0.203\right)\;s$ for dysphagia patients. The timing of BI/EMG-based DoSO is more precise because the standard deviation of the EMG-based DoSO is more than twice as high for healthy subjects and dysphagia patients.

The BI/EMG-based DoSO realizes a remarkably improved detection quality compared to EMG-based DoSO according to median $F_{1}$ scores, consisting of improvements of ΔF=0.152 for data series I, ΔF=0.207 for data series II, ΔF=0.203 for data series III, and ΔF=0.257 for data series IV. Thus, on average, the BI/EMG-based DoSO gained ΔF=0.204$F_{1}$ score points compared to EMG-based DoSO. The highest $F_{1}$ score of the BI/EMG-based DoSO for dysphagia patients is F=0.556[0.405] using only data from healthy subjects in data series I, II, and III for the classifier training. The BI/EMG-based DoSO of swallow onsets requires no manual parameter tuning, providing a significant advantage in a clinical application compared to existing approaches.

On average, the BI/EMG-based DoSO determines 56.0% of the performed swallow onsets (S = 0.56) and 50.0% of the detected swallow onsets are triggered by non-swallow events (P = 0.5) for dysphagia patients in Table 5. Therefore, although BI/EMG-based DoSO outperforms EMG-based DoSO, the detection of the onset of swallowing needs to be further improved.

The BI/EMG-based DoSO might perform much better when applied in real biofeedback or triggered FES sessions because the subjects are more concentrated on swallowing and execute fewer movements, provoking a reduced number of false positives in therapy sessions. The experimental setup for the data series IV with dysphagia patients intended to investigate the assessment of swallowing based on BI/EMG measurements. Therefore, the study protocol did not restrict the patients’ behavior beyond the monitored swallows. Therefore, the data is likely not representative for biofeedback or triggered FES sessions in dysphagia therapy. In clinical practice, most patients learn to reduce undesired movements and evolve toward healthier swallowing patterns, probably enhancing the performance of the BI/EMG-based DoSO.

The lower performance for dysphagia patients likely originates from the reduced EMG activation that causes a smaller contraction of swallow-related muscles and decreases the elevation of the larynx and the depth of BI valleys. Therefore, the reduced swallow ability might impede the separation of swallow onsets from head and jaw movements. Additionally, dysphagia patients sometimes use head movements to initiate swallows, which blurs the difference between swallow onsets and head movements.

The study has the following additional limitations. The ground truth relies on manual annotations of swallow onsets marked by only one expert. Employing several experts leads to more reliable ground truth and enhances the reliance of the results. The timing analysis depends on times of swallow onsets manually marked by an expert based on BI and EMG data. Therefore, the ground truth tends to be more in favor of the BI/EMG-based DoSO.

The threshold $\theta^{\mathrm{PS}}$ for the BI-based preselection of swallow onsets determines the mean detection $\mu^{d}$ of the BI/EMG-based DoSO. Realizing a desired mean detection delay of 0.039 s demands a threshold $\theta^{\mathrm{PS}}$ of 0.18 Ω. The mean detection delay of the EMG-based DoSO was limited to the mean detection delays of the BI/EMG-based DoSO to ensure comparable results for both approaches. Therefore, the results of the EMG-based and BI/EMG-based DoSO depend on the choice of the desired detection delay.

The reported DoSO performances depend on the selected pre-processing of the EMG and BI signals. In this work, no systematic investigation of the filter types and filter parameters was performed, as the complexity of such an optimization is extremely high. It is expected that a future optimization of the pre-processing could further increase DoSO performance. With more training data and sufficient computing power on the intended wearables for the use of the algorithm, deep learning methods could also be used in the future to better tackle this classification problem.

Another limitation of this study is that the involved healthy subjects are much younger in comparison to the patients with dysphagia. We expect that swallow performance will degrade in general with age, leading to less EMG activity as well as smaller and less steep BI changes during swallows. An age-matched control group would certainly have been better for this study. Future work should investigate and compare the performance of DoSO also explicitly for different patient populations, like geriatric patients, ENT-cancer patients, and patients with stroke or Parkinson’s disease.

## 5. Conclusions

The article investigated a threshold method based on EMG data and a machine learning approach to detect swallow onsets in BI and EMG data. The evaluation employs a comprehensive database that included measurements of 41 dysphagia patients. The ground truth utilizes manual annotations of swallow onsets marked by an expert. The $F_{1}$ score rated the detection performance and the standard deviation of the detection delays measures the timing of the detected swallow onsets.

The BI/EMG-based DoSO outperforms the EMG-based DoSO with an $F_{1}$ score of 0.546 compared to 0.289 for dysphagia patients. Further, the standard deviation of the detection delays is reduced by a factor of two from 0.203 s for the EMG-based DoSO to 0.1 s for the BI/EMG-based DoSO with similar mean values of the detection delay. Therefore, the timing of the BI/EMG-based DoSO is more precise compared to the EMG-based DoSO. The article presents the first analysis of the detection performance and timing of an EMG-based DoSO, based on comprehensive data from both healthy subjects and dysphagia patients.

The BI/EMG-based DoSO has the potential to improve biofeedback and triggered FES in dysphagia therapy. The results are still preliminary due to the small number of patients examined. Further studies are required with more patients who suffer from swallowing disorders due to various conditions. All developed methods of BI/EMG-based DoSO are purely causal, which makes a future real-time implementation likely. The methods omit individual parameter tuning. A long-term goal is to employ the DoSO on wearables to enable home care applications for dysphagia management. Such applications might be used in therapy sessions or in everyday life.

## Figures and Tables

**Table 1:** The main properties of data series I to IV. Data series I to III contain data from healthy subjects, and data series IV consists of measurements from dysphagia patients. The table contains the number and sex of subjects, the mean age of the subjects with the standard deviation in brackets, the number of swallows, the cumulative duration of the measurements, and a brief commentary on the research intention for data series I to IV.

Data Series	Subjects	Age	Swallows	Duration	Commentary
I	20 (8♀, 12♂)	30.5 (7.7)	965	3.68 h	movements
II	15 (4♀, 11♂)	29.0 (4.5)	2044	7.10 h	repeatability
III	9 (7♀, 2♂)	38.6 (9.4)	130	0.24 h	investigators
IV	41 (15♀, 26♂)	63.4 (13.8)	704	2.49 h	patients

**Table 2:** The hyperparameter space for the random forest classifier, consisting of the optimization method, the hyperparameter sampling distribution, the hyperparameter name, and the range of the hyperparameter.

Optimization	Hyperparameter	Distribution	Range
grid search	**weight**_1_	n.a.	[0.5,3]
random search	**min_impurity_decrease**	uniform distribution	[0.0,0.001]
random search	**ccp_alpha**	uniform distribution	[0.0,0.00125]
random search	**min_weight_fraction_leaf**	uniform distribution	[0.0,0.0025]
random search	**max_samples**	uniform distribution	[0.65,0.85]

**Table 3:** Scores to evaluate the BI-based preselection of swallow onsets with the parameters: $\theta^{\mathrm{PS}}=0.18\;\Omega$, $\theta^{\mathrm{LA}}=0.5\;s$, and $\theta^{\mathrm{skip}}$ = 1.0 s. The mean detection delay $\mu^{d}$, the standard deviation $\sigma^{d}$ of the detection delay, the ratio of swallow to non-swallow events Υ, and the sensitivity $S^{\mathrm{PS}}$ are presented for data series I, II, III, and IV. The last column contains the average scores calculated from the columns for data series I, II, III, and IV.

Data Series	$S^{\mathrm{PS}}$ [-]	Υ [-]	$\mu^{d}\;\left(\sigma^{d}\right)$ [s]
I	0.993	0.139	0.031 (0.068)
II	0.998	0.256	0.044 (0.076)
III	0.977	0.199	0.048 (0.056)
IV	0.98	0.114	0.033 (0.1)
Mean I, II, III, IV	0.992	0.191	0.039 (0.079)

**Table 4:** Test results for EMG-based detection of swallow onsets, concerning the highest median $F_{1}$ score. The table presents the median and interquartile range of the sensitivity, precision, and $F_{1}$ score for data series I, II, III, and IV. A LOSO cross-validation determined the optimal parameters with respect to the maximal $F_{1}$ score while limiting the mean detection delay to the mean detection delay of the BI/EMG-based DoSO. The last column displays $\mu^{d}$ and $\sigma^{d}$, describing the timing of the EMG-based DoSO.

Data Series	Sensitivity [-]	Precision [-]	$F_{1}$ Score [-]	$\mu^{d}\;\left(\sigma^{d}\right)$ [s]
I	0.84 [0.131]	0.425 [0.147]	0.553 [0.135]	0.019 (0.172)
II	0.759 [0.235]	0.603 [0.463]	0.619 [0.199]	0.039 (0.195)
III	0.625 [0.292]	0.455 [0.509]	0.529 [0.127]	0.022 (0.182)
IV	0.5 [0.678]	0.197 [0.484]	0.289 [0.496]	0.018 (0.203)

**Table 5:** Test results for BI/EMG-based detection of swallow onsets concerning the median $F_{1}$ maximum. The table presents the median and interquartile range of the sensitivity, precision, and $F_{1}$ score for data series I, II, III, and IV regarding the maximal $F_{1}$ score reached by the hyperparameter optimization using a nested LOSO cross-validation. The last column displays $\mu^{d}$ and $\sigma^{d}$, describing the timing.

Data Series	Sensitivity [-]	Precision [-]	$F_{1}$ Score [-]	Specificity [-]	$\mu^{d}\;\left(\sigma^{d}\right)$ [s]	${\mathrm{weight}}_{1}$
I	0.827 [0.129]	0.656 [0.253]	0.705 [0.191]	0.945 [0.064]	0.031 (0.068)	2.0
II	0.875 [0.139]	0.75 [0.183]	0.826 [0.094]	0.956 [0.139]	0.044 (0.076)	0.5
III	0.698 [0.277]	0.846 [0.159]	0.732 [0.184]	0.943 [0.034]	0.048 (0.056)	1.5
IV	0.56 [0.482]	0.5 [0.321]	0.546 [0.405]	0.943 [0.084]	0.033 (0.1)	2.0

**Table 6:** Test results for BI/EMG-based detection of swallow onsets concerning the median $F_{1}$ maximum with mixed data for classifier training. The first row presents the classification performance for training data extracted from data series I to III and test data extracted from data series IV. The second row contains the classification performance for training data extracted from data series I to IV and testing with the data of one subject of data series IV excluded from the training.

Training	Test	Sensitivity [-]	Precision [-]	$F_{1}$ Score [-]	Specificity [-]	${\mathrm{weight}}_{1}$
I, II, III	IV	0.544 [0.539]	0.556 [0.458]	0.556 [0.405]	0.949 [0.067]	1.5
I, II, III, IV	IV	0.603 [0.457]	0.455 [0.333]	0.518 [0.378]	0.925 [0.083]	1.0

## Data Availability

The data presented in this study are available upon reasonable request from the corresponding author.

## Abbreviations

The following abbreviations are used in this manuscript:

BI	Bioimpedance
EMG	Electromyography
eEMG	envelope of Electromyography
DoSO	Detection of Swallow Onsets
FES	Functional Electric Stimulation
FN	False Negatives
FP	False Positives
LOSO	Leave-One-Subject-Out
KDE	Kernel Density Estimation
RF	Random Forest
RT	Reference Times
tEMG	trend in Electromyography
TI	Time of Interest
TN	True Negatives
TP	True Positives
VFSS	Videofluoroscopic Swallowing Study

## Appendix A. Overlap Estimation and Results of Feature Relevance Optimization

This work utilizes an overlap estimation of distributions for feature relevance optimization.

Estimating the overlap for features with N∈N discrete integer values $x_{i,j}\in \left\{1,\ldots ,N\right\}$∀i and j=const. interprets the discrete feature values as bins of the distributions $f_{A}\left(n\right)$ and $f_{B}\left(n\right)$. Given a data set of true samples *A* and a data set of false samples *B*, the probability estimates

(A1) $$\begin{array}{c} p_{A}\left(n\right)=\frac{\left|\{A|x_{i}=n\}\right|}{\left|A\right|} \end{array}$$

(A2) $$\begin{array}{c} p_{B}\left(n\right)=\frac{\left|\{B|x_{i}=n\}\right|}{\left|B\right|} \end{array}$$

with n=1,2,…,N of a feature value *n* being the quotient of the number of features with the value *n* and the number of features in the data set. The operator |.| returns the number of elements in a set. Summing up the minimum of the probability estimates $p_{A}\left(n\right)$ and $p_{B}\left(n\right)$ yields the overlap coefficient

(A3) $$\begin{array}{c} \hat{\eta }\left(A,B\right)=\sum_{n=1}^{N}\min\left[p_{A}\left(n\right),p_{B}\left(n\right)\right] \end{array}$$

for the given data. A smaller value of the overlap coefficient coincides with a larger difference in the probability distributions, indicating a higher feature relevance.

Extending this method for real-valued feature values $x_{i,j}\in R$∀i and index j=const. with distinct values requires the definition of (N+1) borders $x_{n}^{\mathrm{bin}}$ to split the data $D=\{\left(y_{i},x_{i},s_{i}\right)\}$ with i=1,2,…,I into N∈N subsets $D_{n}^{\mathrm{bin}}$. Defining an equal sample number $N^{\mathrm{bin}}=\left|D_{n}^{\mathrm{bin}}\right|\ll I$ for the first N−1 subsets yields a rule to determine the borders and number of bins. The first border $x_{1}^{\mathrm{bin}}\;=\;\min\left(x_{i,j}\right)$ is the minimal feature value, and the greatest border $x_{N+1}^{\mathrm{bin}}\;=\;\max\left(x_{i,j}\right)$ is equal to the maximal feature value. Setting the remaining borders $x_{n}^{\mathrm{bin}}$ with n=2,…,N follows a simple rule

(A4) $$\begin{array}{c} N^{\mathrm{bin}}=\left|D_{n}^{\mathrm{bin}}\right|=\left|\left\{D|x_{n}^{\mathrm{bin}}<x_{i,j}<x_{n+1}^{\mathrm{bin}}\right\}\right|. \end{array}$$

The last subset $D_{N}^{\mathrm{bin}}$ with the borders $x_{N}^{\mathrm{bin}}$ and $x_{N+1}^{\mathrm{bin}}$ includes the remaining $\left[I-N^{\mathrm{bin}}\;\cdot \;\left(N-1\right)\right]$ samples. Computing the probabilities

(A5) $$\begin{array}{c} p_{A}\left(n\right)=\frac{|A_{n}|}{\left|A\right|}\;\mathrm{with}\;A_{n}=\left\{D_{n}^{\mathrm{bin}}|y_{i}=1\right\} \end{array}$$

(A6) $$\begin{array}{c} p_{B}\left(n\right)=\frac{|B_{n}|}{\left|B\right|}\;\mathrm{with}\;B_{n}=\left\{D_{n}^{\mathrm{bin}}|y_{i}=0\right\} \end{array}$$

with n=1,2,…,N yields the overlap of real-valued features using (A3).

The overlap estimation utilizes $N^{\mathrm{bin}}=50$ samples per bin for real-valued features with the indices j=1,2,3,4,5,6,9,10. The features with the indices j=7,8,11,12 are integer-valued.

Table A1 presents the parameters $\Delta_{j}$ and $N_{j}$ for the feature relevance optimization (cf. Section 2.6.4) together with the obtained results.

**Table A1:** Overview of sample increment $\Delta_{j}$, the number $N_{j}$ of maximal possible increments, the sampling frequency $f_{T}$, the obtained number of optimal samples $M_{j}^{\mathrm{opt}}$, the corresponding optimal time interval $t_{j}^{\mathrm{opt}}$, and optimal overlap estimate ${\hat{\eta }}_{j}^{\mathrm{opt}}$ for all features j=1,…,12.

Data	*j*	Feature	$\Delta_{j}$	$N_{j}$	$f_{T}$	$M_{j}^{\mathrm{opt}}$	$t_{j}^{\mathrm{opt}}$	${\hat{\eta }}_{j}^{\mathrm{opt}}$
tEMG	1	$\sigma^{\mathrm{tEMG}}$	200	40	4000 Hz	400	0.1 s	78.6%
	2	$\Upsilon^{\mathrm{tEMG}}$	200	40	4000 Hz	6000	1.5 s	82.1%
	3	${\hat{\sigma }}^{\mathrm{tEMG}}$	600	20	4000 Hz	1200	0.3 s	80.4%
BI	4	$\sigma^{\mathrm{BI}}$	5	40	100 Hz	30	0.3 s	63.1%
	5	$\Upsilon^{\mathrm{BI}}$	5	40	100 Hz	190	1.9 s	74.5%
	6	${\hat{\sigma }}^{\mathrm{BI}}$	15	20	100 Hz	75	0.75 s	63.8%
	7	${\bar{\sigma }}^{\mathrm{BI}}$	15	20	100 Hz	75	0.75 s	78.4%
	8	${\underline{\sigma }}^{\mathrm{BI}}$	15	20	100 Hz	45	0.45 s	82.6%
EMG	9	AAC	100	40	4000 Hz	300	0.075 s	39.9%
	10	$\delta^{\mathrm{EMG}}$	100	40	4000 Hz	1700	0.425 s	38.4%
	11	${\bar{\sigma }}^{\mathrm{EMG}}$	600	20	4000 Hz	7200	1.8 s	40.8%
	12	${\underline{\sigma }}^{\mathrm{EMG}}$	600	20	4000 Hz	1800	0.45 s	60.1%
